# Hsp22 Deficiency Induces Age-Dependent Cardiac Dilation and Dysfunction by Impairing Autophagy, Metabolism, and Oxidative Response

**DOI:** 10.3390/antiox10101550

**Published:** 2021-09-29

**Authors:** Wenqian Wu, Xiaonan Sun, Xiaomeng Shi, Lo Lai, Charles Wang, Mingxin Xie, Gangjian Qin, Hongyu Qiu

**Affiliations:** 1 Center for Molecular and Translational Medicine, Institute of Biomedical Science, Georgia State University, Atlanta, GA 30303, USA; wwenqian@gsu.edu (W.W.); xsun13@gsu.edu (X.S.); xshi8@student.gsu.edu (X.S.); lo.lai@nih.gov (L.L.); 2 Department of Ultrasound Medicine, Union Hospital, Tongji Medical College, Huazhong University of Science and Technology, Wuhan 430030, China; xiemx@hust.edu.cn; 3 Center for Genomics, Department of Basic Sciences, School of Medicine, Loma Linda University, Loma Linda, CA 11175, USA; chwang@llu.edu; 4 Department of Biomedical Engineering, School of Medicine and School of Engineering, University of Alabama at Birmingham, Birmingham, AL 35294, USA; gqin@uab.edu

**Keywords:** Hsp22, cardiomyopathy, cardiac dysfunction, autophagy, metabolism, oxidative stress

## Abstract

Heat shock protein 22 (Hsp22) is a small heat shock protein predominantly expressed in skeletal and cardiac muscle. Previous studies indicate that Hsp22 plays a vital role in protecting the heart against cardiac stress. However, the essential role of Hsp22 in the heart under physiological conditions remains largely unknown. In this study, we used an Hsp22 knockout (KO) mouse model to determine whether loss of Hsp22 impairs cardiac growth and function with increasing age under physiological conditions. Cardiac structural and functional alterations at baseline were measured using echocardiography and invasive catheterization in Hsp22 KO mice during aging transition compared to their age-matched wild-type (WT) littermates. Our results showed that Hsp22 deletion induced progressive cardiac dilation along with declined function during the aging transition. Mechanistically, the loss of Hsp22 impaired BCL-2–associated athanogene 3 (BAG3) expression and its associated cardiac autophagy, undermined cardiac energy metabolism homeostasis and increased oxidative damage. This study showed that Hsp22 played an essential role in the non-stressed heart during the early stage of aging, which may bring new insight into understanding the pathogenesis of age-related dilated cardiomyopathy.

## 1. Introduction

Heat shock proteins (Hsps) comprise a large family of ubiquitously expressed stress proteins that constitute an endogenous stress response system to protect cells against insults such as hypoxia and ischemia. Numerous mutations in genes encoding Hsps have been implicated in many human diseases upon resultant changes in their expression levels and cellular localization [1,2,3]. Heat shock protein 22 (Hsp22), also known as protein kinase H11, HspB8, or αC-crystallin, is one of the small Hsps predominantly expressed in the heart and skeletal muscles [4,5]. A dramatic upregulation of cardiac Hsp22 expression has been observed in well-characterized animal models of cardiovascular disease and patients diagnosed with various forms of cardiac disorders, including acute and chronic myocardial ischemic injuries and pressure overload-induced cardiac hypertrophy [6,7]. More so, our previous studies also demonstrated that Hsp22 overexpression could protect the heart against ischemic injury [8]. Reciprocally, Hsp22 deletion accelerates cardiac dysfunction and ventricular remodeling leading to heart failure (HF) in a pressure overload-induced hypertrophic mouse heart [9]. These studies together indicate that Hsp22 plays a vital role in providing myocardial protection in a stressed heart. Thus, most of the current studies about Hsp22 are focused on its immediate responses to acute stress in cultured cardiomyocytes or primary animal models of heart diseases. Still, the essential role of Hsp22 in the heart under physiological conditions remains largely unknown.

Although Hsp22 was initially known for possessing intrinsic protein kinase activity, emerging evidence has shown that Hsp22 predominantly exhibits molecular chaperone-like activity, enabling functional diversity through interactions with many proteins without employing its kinase activity [2,10,11]. Despite a lack of full knowledge on these protein-protein interactions, recent studies have revealed that molecular functions of Hsp22 are highly dependent on BCL-2–associated athanogene 3 (BAG3) [12,13,14], which is a co-chaperone protein abundantly expressed in the heart. Hsp22 has been shown to directly bind to BAG3 through the BAG3 intermediate domain, and BAG3 deletion eliminates Hsp22 expression in cardiomyocytes [15], indicating BAG3-dependent Hsp22 expression. BAG3 has been reported to act in concert with Hsp22 to induce autophagic degradation of mechanically damaged cytoskeleton components. Mutated or deficient BAG3 expression has been repeatedly associated with the development of human cardiomyopathy [16,17]. However, little is known about the potential effects of Hsp22 on BAG3 expression and the subsequent pathophysiological changes in the normal heart.

The mammalian heart is one of the most energy-consuming organs in the body, and normal cardiac function relies highly on timely and efficient cardiac metabolism. The heart utilizes various metabolites for energy production, such as fatty acids (FA), glucose, ketone bodies, lactate, pyruvate, and amino acids. Among all these metabolic substrates, the heart mainly utilizes long-chain FA (LCFA), accounting for 50–70% of its overall fuel metabolism [18]. Although glucose serves as a fuel of choice for many tissues and organs due to its fast breakdown to pyruvate and energy, it is less favored by the heart than FAs, which are responsible for producing only about 30% of the overall fuel. Metabolic remodeling in the heart, by definition, refers to metabolic changes in selecting, partitioning, and coordinating different substrates in the process of energy production to confer adaptive or maladaptive responses to various triggering situations. Maladaptive cardiac metabolic remodeling has been implicated in the pathogenesis of many cardiovascular problems [19,20], such as HF [21,22]. In addition, progressive metabolic remodeling occurs during cardiac aging and age-related cardiomyopathy, which is inherently triggered and advanced by a series of alterations in autophagy and oxidative stress. Although Hsp22 is functionally active for defending various pathological processes in the stressed heart, its essential biologic effects on aging-related cardiac changes regarding metabolism, autophagy, and oxidative stress have not been explored.

Based on previous studies, we hypothesized that Hsp22 is fundamentally necessary for maintaining normal cardiac function under physiological conditions during aging, whereas the deletion of Hsp22 compromises such protection in cardiac aging, leading to myocardial dysfunction and cardiomyopathy. To test this hypothesis, we used a previously generated Hsp22 knockout (KO) mouse model to determine whether the deletion of Hsp22 impairs cardiac growth and function under physiological conditions during the aging transition. We performed a time-course study to determine the impact of Hsp22 deletion on cardiac morphology and contractile function in Hsp22 KO mice at baseline with an increasing age, by using echocardiography and invasive hemodynamic measurements followed by data comparison with age-matched wild-type (WT) control mice. We also investigated the underlying molecular basis involving cardiac autophagy, metabolic remodeling, and oxidative stress. Our study provides novel evidence for the essential role of Hsp22 in the non-stressed heart during the aging transition and the associated mechanisms underpinning the development of cardiac dysfunction and cardiomyopathy induced by Hsp22 deficiency. It also brought new insights to better understand the pathogenesis of age-associated heart diseases.

## 2. Materials and Methods

### 2.1. Animal Models

An Hsp22 KO mouse(C57/6J) was generated as described previously [9], in which Hsp22 expression was knocked out compared with their litter-matched WT mice. 16- to 54-week-old Hsp22 KO mice, males and females, and their WT littermates were studied.

### 2.2. Echocardiography

M-mode echocardiography: Echocardiography was performed using a Vevo 3100 high-resolution micro-ultrasound system (FUJIFILM Visual Sonics Inc., Toronto, ON, Canada) under anesthesia with 2% isoflurane, as previously described [23]. The heart rates of all mice were maintained above 450 bpm while being imaged. The M-mode tracing was used to measure left ventricle (LV) structural and functional parameters as we did previously [23] and detailed in the below results.

Two-dimensional speckle tracking echocardiography (2D-STE): 2D-STE was used to assess myocardial deformation. Both LV long-axis and short-axis B-mode images with a frame rate ≥200 frames/second were collected and digitally stored in cine loops. The strain was used to assess relevant changes in myocardial fiber length by measuring both longitudinal and circumferential planes. Strain rate (SR) was calculated to reflect myocardial deformation over time. The long- and short-axis views were used to determine global longitudinal and circumferential strains (GLS and GCS) and their maximal deformation rates (LSR and CSR) during systole, respectively. The “reverse peak” algorithm of the Vevo Strain software was utilized to measure longitudinal and circumferential reverse strain (rLS and rCS) during diastole and their maximal reverse strain rate (LrSR and CrSR). All measurements were analyzed using Vero Strain software (Vevo Lab 3.2.6).

### 2.3. Hemodynamics Using a Millar Pressure Catheter

A Millar catheter-tip micromanometer catheter (SPR-671; Millar Instruments) connected to a power laboratory system (AD Instruments, Castle Hill, Australia) was used for the hemodynamic analysis as described previously [24,25,26], and detailed in results. Contractility index and LV systolic wall stress (LVSWS) were calculated as follows: contractility index(s)=LVESP/Max dp/dt; LVSWS (in kdyn/cm^2^) = (1.35 × LVESP × LVESD)/[4 × LVPWs × (1 + LVPWs/LVESD)] [27].

### 2.4. Histology

After carotid catheterization, mice were euthanized by carbon dioxide (CO_2_) inhalation, and heart tissues were collected for ex vivo experiments. The mouse hearts and LVs were weighted and normalized by tibia length (TL).

Mid-ventricular cross-sections were fixed in 4% paraformaldehyde and embedded in paraffin. Sections at a thickness of 4 μm were subjected to xylene and alcohol for dehydration. The cross-sectional area (CSA) was determined using wheat germ agglutinin (WGA)-staining (Invitrogen, W849) and measured using Image-J as previously performed [9,25]. Cardiac fibrosis was measured with picrosirius red staining (PSR) staining and presented by the percentage of fibric area [9,25].

Oxidative stress markers, including 8-hydroxy-2′-deoxyguanosine (8-OHdG), 4-hydroxynonenal (4HNE), and malondialdehyde (MDA), were measured using immunofluorescent staining [23]. The slides were subjected to citrate buffer and blocked with blocking buffer. Slides were incubated with primary antibodies at 4 °C overnight. Alexa Fluor 488 and 568 goat anti-mouse IgG (Invitrogen, A-11029 and A-11004, 1:500 dilution) were used as secondary antibodies, incubated at room temperature for 1 h. The slices were mounted with DAPI ready-made solution with antifade (Sigma-Aldrich, MBD0020) for fluorescence microscopy. Primary antibody information was shown in Table 1.

### 2.5. Protein Extraction and Western Blot

Total protein was extracted from LV tissues and then processed using western blot followed by detection with the LI-COR Odyssey infrared imaging system (LI-COR Biosciences, Lincoln, NE, USA) as described previously [23,26]. GAPDH or β-tubulin was used for loading control. Primary antibody information was also shown in Table 1.

### 2.6. ATP Assay

ATP production was measured using the bioluminescence assay with ATP Determination Kit (Thermo Fisher Scientific, A22066). All the procedures were followed by the manufacturer’s instructions. Briefly, mice heart tissues were homogenized with lysis buffer. Then protein solution was added in reaction buffer containing 1 mM dithiothreitol, 0.5 mM D-luciferin and 12.5 µg/mL firefly luciferase. The results were determined by luminescence after a 15 min incubation (BioTek SYNERGY/LX multi-mode reader) and then normalized to the amount of the proteins (valued as nM/mg proteins).

### 2.7. Statistical Analysis

All values were presented as mean ± SEM. Differences between experimental groups were determined using the *t*-test or two-way ANOVA as previously performed [9,23], and detailed in figure legends. *p* < 0.05 was considered statistically significant.

## 3. Results

### 3.1. Hsp22 Deletion Induces a Progressive Cardiac Dilation with Increasing Age

To explore the undetermined role of Hsp22 in the heart under physiological conditions, we generated a homozygous Hsp22 KO mouse model in which Hsp22 expression was entirely absent [9]. Age-matched WT littermates were used as a control. We performed a time-course study to observe the initial and progressive cardiac alterations in these mice at three-time points, young (16.7 ± 0.23 weeks), middle-aged (33.08 ± 0.27 weeks), and older (52.72 ± 0.28 weeks), which were equivalent to humans aged 26–30, 40–44 and 58–62 years old, respectively (https://animalyearstohumanyears.com/ accessed on 1 September 2021). Echocardiography was used to measure cardiac morphological and structural changes in these mice at different ages.

**Figure 1:**
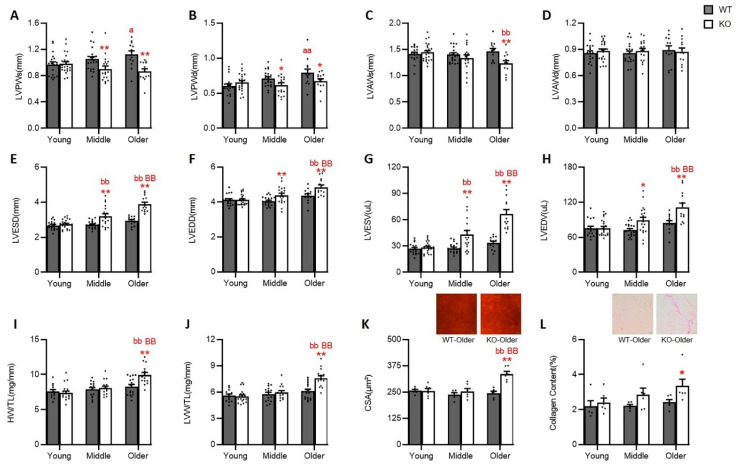
Loss of Hsp22 progressively induced cardiac dilation with the increasing age. (**A**–**D**): The left ventricle (LV) end-systolic and end-diastolic wall thickness of posterior (LVPWs (**A**), LVPWd (**B**)), and anterior (LVAWs (**C**) and LVAWd (**D**)) of Hsp22 knockout (KO) and their age-matched wild-type (WT) mice. (**E**–**H**): LV end-systolic and end-diastolic internal diameters (LVESD (**E**), LVEDD (**F**)), and volumes (LVESV(**G**), and LVEDV(**H**)). (**I**,**J**): Heart weight/tibial length ratio (HW/TL) and left ventricle weight/tibial length ratio (LVW/TL). (**K**,**L**): Histological quantification of the cross-sectional area (CSA) of cardiomyocytes by wheat germ agglutinin staining and relative myocardial fibrotic area (MFA) by Picro Sirius Red staining. * *p* < 0.05, ** *p* < 0.01, vs. WT; a: *p* < 0.05, aa: *p* < 0.01, vs. young WT; bb: *p* < 0.01 vs. young KO; BB: *p* < 0.01 vs. middle age KO. *N* = 13–23/group (male 6–14/group and female 4–15/group) for (**A**–**H**). N = 13–19/group (male 5–10/group and female 5–10/group) for (**I**,**J**). *N* = 6/group for (**K**,**L**). Data are shown as mean ± SEM, two-way ANOVA was used in (**A**–**J**), and *t*-test was used in (**K**,**L**).

We first compared age-related cardiac alterations in the LV chamber shape and size among the WT mice. As shown in Figure 1A,B, WT mice exhibited progressively increased LV posterior wall thickness at end-systole and end-diastole (LVPWs and LVPWd), reaching statistical significance in the older WT mice compared to the WT young mice. There is no significant difference in wall thickness between the middle-aged and older WT mice (Figure 1A,B) (*p* > 0.05 vs. middle-aged). These data indicate a physiological LV wall thickening from young to middle age. Still, this physiological cardiac growth is much less evident during the transition from middle age to older age in WT mice. No significant difference was detected in the LV anterior wall thickness (LVAWs and LVAWd) (Figure 1C,D) nor in LV chamber internal dimensions at end-systolic and end-diastolic (LVESD and LVEDD) (Figure 1E,F), or volumes (LVESV and LVEDV) (Figure 1G,H) through the three phases of aging in the WT mice. These data indicated that the chamber size remains unchanged across the three age points in the WT mice.

Interestingly, age-related LV wall thickening was not observed in the Hsp22 KO mice (Figure 1A,B). On the contrary, LVAWs dramatically decreased in the older KO mice than the young KO mice (Figure 1C). LVESD and LVEDD were significantly increased in the older KO mice compared to the young and middle-aged KO mice (Figure 1E,F), resulting in corresponding remarkably enlarged LV volumes (Figure 1G,H). These data indicated that the Hsp22 KO mice developed progressive LV dilation with increasing age. Notably, the LV chamber size at end-systole (LVESD and LVESV) showed an even earlier increase in the middle-aged KO mice vs. the young KO mice (Figure 1E,G), which may imply a gradual weakening in contractility starting at this age.

We then compared cardiac shape and size between the Hsp22 KO and WT mice at each of the three age points. There was no significant difference at the young age between the Hsp22 KO and WT mice regarding LV wall thickness, diameters, and volumes (Figure 1A–H). These data were consistent with our previous observations [9]. However, the middle-aged Hsp22 KO mice revealed a significantly cardiac dilation compared to their age-matched WT control, shown as decreased LV wall thickness (Figure 1A,B) along with increased LV diameters and chamber volumes (Figure 1E–H). The observed cardiac dilation was further exacerbated in the older KO mice vs. the WT mice (Figure 1A,B,E–H).

In addition to the above measurements from the echocardiography, we also compared heart weights between the KO and WT mice at different ages. The total heart weight (HW) and left ventricular weight (LVW) were measured and normalized to their TL. No significant differences in HW/TL or LVW/TL were found in the WT mice throughout the three aging periods. On the contrary, HW/TL and LVW/TL were significantly increased in older the KO mice, exhibiting a remarkably larger heart along with an increased LV weight compared to the young and middle-aged KO mice (Figure 1I,J).

Histological examinations were performed to detect changes in cardiomyocyte size and cardiac fibrosis between the Hsp22 KO and WT groups during the aging transition. The results showed that cardiac myocyte size, represented by CSA, was significantly increased in the older Hsp22 KO mice compared to their age-matched WT mice (Figure 1K). Cardiac fibrosis, represented by the percentage of fibrotic area, was also significantly increased in the older Hsp22 KO mice than in the older WT mice (Figure 1L).

### 3.2. Hsp22 Deletion Impaired Cardiac Function with the Increasing Age

**Figure 2:**
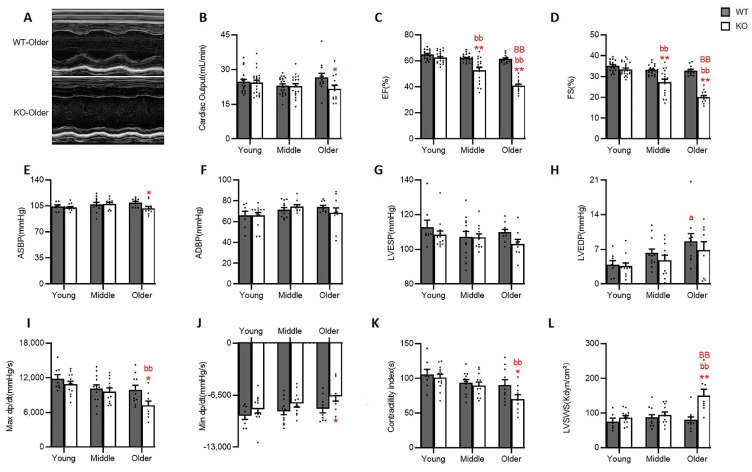
Hsp22 deletion attenuated cardiac function with the increasing age. (**A**): Representative M-mode images of left ventricle (LV) from WT and Hsp22 KO older mice. (**B**–**D**): Cardiac functional indexes by M-mode echocardiography, including cardiac output (**B**), LV-ejection fraction (EF)(**C**), and fractional shortening (FS) (**D**). (**E**–**J**): Quantitated hemodynamic data by an invasive Millar pressure catheter, including aortic systolic (ASBP) (**E**) and diastolic blood pressure (ADBP) (**F**), left ventricular (LV) end-systolic (LVESP) (**G**) and diastolic pressure (LVEDP) (**H**), the maximum and minimum derivative of pressure over time (max dp/dt (**I**), and min dp/dt (**J**)). (**K**): Contractility index calculated using catheter data and (**L**): LV systolic wall stress (LVSWS) calculated using both echo and catheter measurements. * *p* < 0.05, ** *p* < 0.01, vs. WT; a: *p* < 0.05, vs. young of WT group; bb: *p* < 0.01 vs. young of KO group; BB: *p* < 0.01 vs. middle of KO group. *N* = 13–23/group (male 6–14/group and female 4–15/group) for (**B**–**D**). *N* = 13–19/group (male 5–10/group and female 5–10/group) for (**E**–**L**). Data are shown as mean ± SEM, two-way ANOVA was used for all data analysis.

We further detected the impact of Hsp22 deletion on cardiac function in the unstressed heart with the increasing age by combining echocardiography as represented in Figure 2A, with invasive hemodynamic measurements using a Millar pressure catheter.

We first used the classic M-mode echo to detect changes in cardiac contractile function in WT during this aging transition period. There was no significant functional difference in the WT mice at the three different ages, in terms of cardiac output (CO) (Figure 2B), ejection fraction (EF) (Figure 2C), and fractional shortening (FS) (Figure 2D). Next, we used a pressure-tipped catheter to detect aortic blood pressure and hemodynamic changes in the WT mice (Figure 2E–J). No significant differences were seen in aortic systolic and diastolic blood pressure (ASBP and ADBP) (Figure 2E,F), LV end-systolic pressure (LVESP) (Figure 2G), and or maximum and minimum derivatives of pressure over time (maxdp/dt and mindp/dt) (Figure 2I,J). However, LV end-diastolic pressure (LVEDP) was progressively elevated with increasing age and reaching statistical significance in the older WT mice compared to the young WT mice (Figure 2H). Two integrated functional indicators, contractility index and LVSWS, were also adopted to assess comprehensive cardiac function. The results showed no significant difference among the WT mice at different ages (Figure 2K,L). These data together suggested that cardiac function remained relatively unchanged in WT mice during the aging transition.

In contrast, Hsp22 deletion resulted in an age-dependent impairment in LV contractile function, as evidenced by a dramatically progressive decline in EF and FS, starting from middle age and becoming more significant in the older KO mice, compared to young KO mice (Figure 2C,D). In addition, the older KO mice exhibited a remarkable reduction in max dp/dt (Figure 2I), and contractility index (Figure 2K) accompanied by a dramatic increase in LVSWS (Figure 2L) compared to the young KO mice.

Cardiac function was also compared between the KO and WT mice at each age point. Despite no significant difference in cardiac function between the young KO and WT mice, the middle-aged Hsp22 KO mice exhibited drastically lowered EF and FS, which was further worsened in the older KO mice than their age-matched WT mice (Figure 2C,D). In addition, ASBP (Figure 2E), max dp/dt, min dp/dt (Figure 2I,J) and contractile index (Figure 2K). were remarkably decreased in the older KO mice vs. their age-matched WT mice, while LVSWS was dramatically increased in the older KO mice vs. WT(Figure 2L).

Together, these results suggested that the LV contractility was significantly impaired in the absence of Hsp22 with increasing age, implying the essential role of Hsp22 in maintaining normal cardiac function, particularly in older age.

### 3.3. Hsp22 Deficiency Impacts Myocardial Deformation Preceding Cardiac Functional Decline

**Figure 3:**
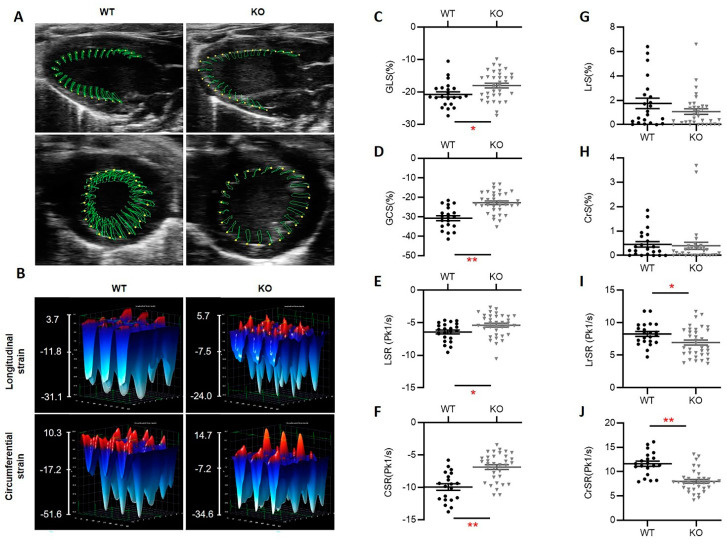
Hsp22 deficiency impaired cardiac muscle deformation preceding the cardiac functional decline. (**A**,**B**): Representative images and illustration of the left ventricular (LV) deformation tracked by the speckle tracking echocardiography (STE) throughout a cardiac cycle (contraction and relaxation phases), longitudinally (upper panel) and circumferentially (lower panel) (**A**), and the three-dimensional strain during three consecutive cardiac cycles for young Hsp22 KO (KO) and young wild-type (WT) mice (**B**), showing the activities of LV contraction (blue/negative values) or relaxation (red/positive values). (**C**–**J**): Quantitative data from STE, including global longitudinal and circumferential strain (GLS (**C**) and GCS (**D**), strain rate (LSR (**E**) and CSR (**F**)), reversed longitudinal and circumferential strain (LrS (**G**), and CrS (**H**)), and reversed strain rate (LrSR (**I**) and CrSR (**J**)). Data are presented as mean ± SEM. * *p* < 0.05, ** *p* < 0.01 vs. WT. *N* = 20–35/group (male, 9–18/group and female, 15–17/group). A *t*-test was used for comparison between two groups.

To further detect early-onset cardiac dysfunction caused by Hsp22 deletion, we adopted 2D-STE, a recently developed and potentially more sensitive quantitative ultrasound technique, to capture cardiac motion and deformation throughout each cardiac cycle. As illustrated in Figure 3A,B, STE recorded the expansion and recoil of the LV wall longitudinally and circumferentially, and myocardial deformation was assessed by directly calculating the myocardial muscle shortening and lengthening. We used the following STE deformation indices for an objective quantification of myocardial activity, including global longitudinal and circumferential strain (GLS and GCS) during systole (Figure 3C,D), their maximal deformation rates represented by strain rates (LSR and CSR) (Figure 3E,F), the reverse longitudinal and circumferential strain during diastole (rLS and rCS) (Figure 3G,H), and their maximal reverse strain rate (LrSR and CrSR) (Figure 3I,J).

We focused on analyzing the echo data collected from young mice whose cardiac function reflected by multiple conventional LV functional parameters such as EF, FS, and contractility index has not yet significantly declined. Our results showed that the STE indices for assessing LV systolic function, including GLS, GCS, LSR, CSR, were dramatically reduced in the young KO mice vs. the young WT mice (Figure 3C–F). The diastolic reverse strain showed a slight decline in the KO mice, but this trend did not reach statistical significance (Figure 3G,H). Moreover, the maximal reverse strain rates, LrSR and CrSR, were significantly lower in KO mice than their age-matched WT mice (Figure 3I,J). These data together indicated that a loss of Hsp22 elicited prominent cardiac muscle weakness and considerably reduced myocardial tissue deformation preceding significant functional impairment, particularly during systole.

### 3.4. Loss of Hsp22 Leads to an Age-Dependent Reduction in Cardiac BAG3 Expression and Impaired Cardiac Autophagy in an Older Age

We further investigated the potential molecular mechanisms involved in the observed physiological alterations in the Hsp22 KO mice. Considering BAG3-dependent Hsp22 expression in cardiomyocytes [15], we first determined whether Hsp22 deletion could conversely affect cardiac BAG3 expression during the aging transition. In the WT mice, the cardiac expression of Hsp22 and BAG3 exhibited a trend of progressively increase during the aging transition, along with a declining trend in the expression of Hsp70. However, BAG3 declined in the Hsp22 KO mice during the aging transition but there were no significant changes in Hsp70 (Figure S1).

**Figure 4:**
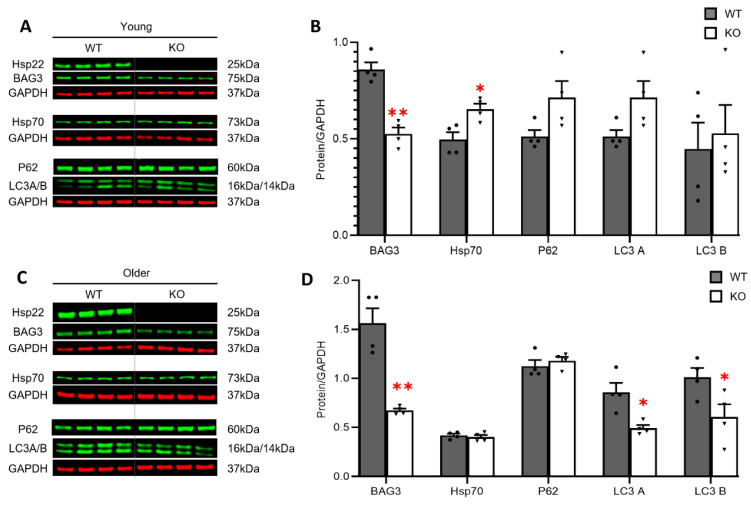
Hsp22 deficiency led to the reduction in BAG3 protein levels and impaired cardiac autophagy. (**A**,**B**): Representative western blots and the quantitated data of the protein expression of BAG3, Hsp70, P62, and LC3 A/B in the hearts of Hsp22 knockout (KO) and wild-type (WT) mice at a young age. (**C**,**D**): Representative western blots and the quantitated data of the same protein expressions in the hearts of KO and WT mice at an older age. GAPDH was used as a loading control. Quantitative relative protein levels were normalized with GAPDH. Data are presented as mean ± SEM. * *p* < 0.05, ** *p* < 0.01 vs. age-matched WT. *N* = 4/group. A *t*-test was used for the comparison between KO and WT groups at each age.

Compared to the age-matched WT mice, the Hsp22 KO mice displayed decreased cardiac BAG3 protein expression in the hearts of the young KO mice (Figure 4A,B), and this reduction became more prominent in the older KO mice (Figure 4C,D). Interestingly, we noticed that Hsp70, a potential co-factor of BAG3/Hsp22, was significantly increased in the hearts of the young Hsp22 KO mice vs. WT but not in the older KO mice (Figure 4A–D).

Since previous research has shown that Hsp22/BAG3 is crucial for forming an autophagy-inducing complex [13,28], we then determined the essential role of Hsp22 in cardiac autophagy by measuring reliable autophagy markers, including an autophagosome cargo protein, ubiquitin-binding protein P62 (P62), also called sequestosome 1 (SQSTM1), and microtubule-associated proteins 1A/1B light chain 3B (LC3s), which are the central proteins in the autophagy pathway. At a young age, P62, and LC3 A and B were slightly increased in the young KO mice vs. WT(Figure 4B), but the increase did not reach a significant difference. In contrast, LC3-A and-B were significantly decreased in the older KO mice vs. the WT mice, indicating impaired autophagy in the heart in older age (Figure 4D).

### 3.5. Deficiency of Hsp22 Interferes Cardiac Metabolic Pathways under Physiological Conditions before Developing Cardiac Dysfunction

Since cardiac function strongly relies on energy supply and the subsequent metabolism, we investigated the initial and progressive impact of Hsp22 deficiency on cardiac metabolisms in young and older mice by examining key molecules involved in FA and glucose metabolic signaling pathways.

**Figure 5:**
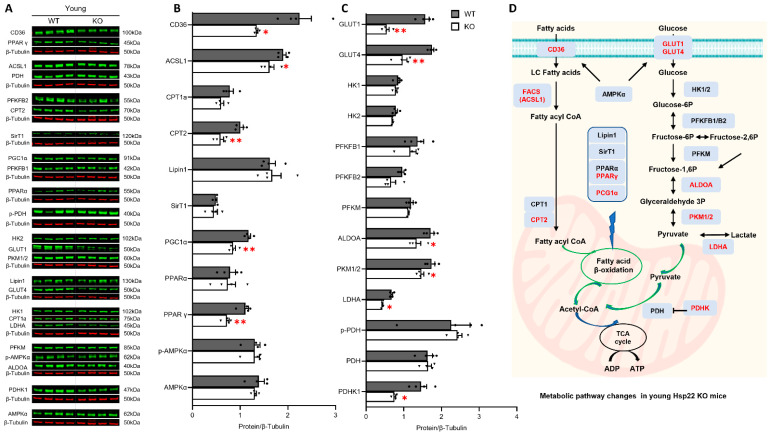
Loss of Hsp22 affects fatty acid (FA) and glucose metabolic pathways at a young age. (**A**): Representative western blots showing the key enzymes in FA and glucose metabolic pathways in the young Hsp22 knockout (KO) and wild-type (WT) mouse hearts. β-tubulin was used as a loading control. (**B**,**C**): Quantitative protein levels normalized by β-tubulin in the young KO and WT groups. (**D**): Schematic diagram shows the impacts of Hsp22 deletion on FA and glucose pathways at a young age (red indicates significantly changed proteins in KO vs. WT mice). Data are presented as mean ± SEM. * *p* < 0.05, ** *p* < 0.01 vs. WT. *N* = 4/group. A *t*-test was used.

We first compared the changes in FA and glucose metabolism between the young Hsp22 KO mice and their age-matched WT using western blotting, as shown in Figure 5A. In terms of FA metabolism, we found that, compared to the WT mice, FA uptake transporter CD36 was significantly decreased in the KO mice (Figure 5B), indicating disturbed cellular FA transport. In addition, long-chain fatty acyl-CoA ligase 1 (ACSL1), which is an isozyme from the CoA synthetase (FACS) family that converts free LCFA into fatty acyl-CoA esters, was decreased in the KO mice (Figure 5B). Carnitine palmitoyltransferase 2 (CPT2), a mitochondrial membrane protein transporting and oxidizing LCFA in the mitochondria, was also remarkedly decreased in the young KO mice (Figure 5B). Furthermore, two proteins regulating FA oxidation (FAO) were found to be downregulated in the young Hsp22 KO mice vs. the WT mice, e.g., peroxisome proliferator-activated receptor gamma (PPARγ) and its coactivator1-alpha (PGC-1α). PPARγ and PGC-1α are two master regulators of mitochondrial oxidative phosphorylation and FAO gene expression. However, no significant difference was detected between the young KO and WT mice in PPAR-alpha (PPARα) expression or AMP-activated protein kinase (AMPK) α subunit (AMPKα) as well as phospho-AMPKα (pAMPKα) (Figure 5B).

We next compared glucose metabolic changes between the young KO mice and the WT mice. Compared to the age-matched WT mice, the expression levels of glucose transporter 1 and 4 (GLUT1 and GLUT4) were significantly decreased in the young KO mice (Figure 5C), indicating compromised glucose transport induced by Hsp22 deficiency in the young KO mice. Three key enzymes that regulate the pace of glycolysis and gluconeogenesis were also significantly reduced in the KO mice, including aldolase A (ALDOA), pyruvate kinase isozymes M1/M2 (PKM1/2), and lactate dehydrogenase A (LDHA). ALDOA functions at the fourth step of the shared pathway of cytosolic glycolysis and gluconeogenesis, catalyzing the reversible conversion of fructose-1,6-bisphosphate (F1,6BP) to glyceraldehyde 3-phosphate (G3P) and dihydroxyacetone phosphate (DHAP). PKM1/2 functions at the final rate-limiting step of glycolysis, catalyzing reversible interconversion of G3P and pyruvate. LDHA catalyzes the interconversion of pyruvate and lactate with synchronized interconversion of NADH and NAD^+^ in anaerobic glycolysis. In addition, mitochondrial pyruvate dehydrogenase kinase1 (PDHK1), which acts to inactivate pyruvate dehydrogenase (PDH), was found to be reduced in the young KO mice vs. the WT mice. However, the expression and phosphorylation of pyruvate dehydrogenase (PDH and p-PDH) have not been evidently affected at this age yet (Figure 5C). We also noticed that Hsp22 deletion did not affect any other proteins in the early steps of glycolysis in young KO mice, such as hexokinase (HK1 and 2) and 6-phosphofructo-2-kinase/fructose-2,6-biphosphatase 2 (PFKFB 1 and 2) (Figure 5C). HK serves as the initial rate-limiting enzyme that converts glucose to glucose-6-phosphate (G6P). PFKFB 1 and 2 indirectly regulate glycolysis homeostasis through mediating fructose-2,6-biphosphate levels. 

As illustrated in Figure 5D, these data together indicate that at an early age, Hsp22 deletion kicked off its impairment on FA metabolism by interfering with FA cellular transport, mitochondrial import, and FAO enzyme gene expression. Glycolysis was also hindered in the young KO mice, reflected by a decreased expression of the glucose transporters and rate-limiting enzymes that regulate glycolysis and gluconeogenesis in the cytosol, but Hsp22 deletion had no significant effect on mitochondrial pyruvate metabolism at this age.

**Figure 6:**
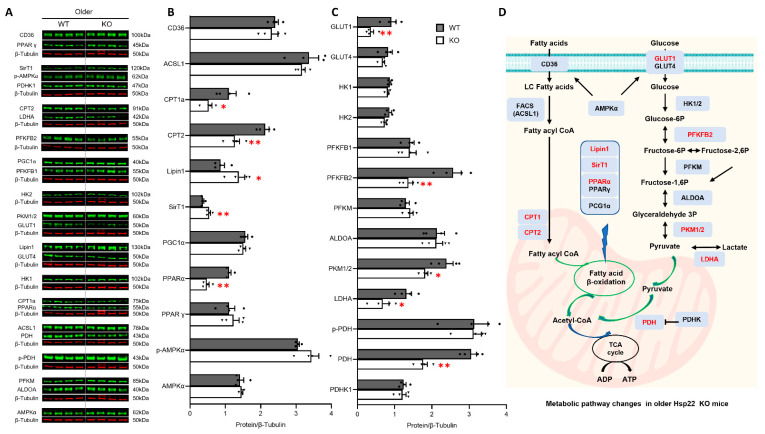
Loss of Hsp22 affects fatty acid (FA) and glucose metabolic pathways at an older age. (**A**): Representative western blots showing the key enzymes in FA and glucose metabolic pathways in the older Hsp22 knockout (KO) and wild-type (WT) mouse hearts. β-tubulin was used as a loading control. (**B**,**C**): Quantitative protein levels normalized by β-tubulin in older KO and WT groups. (**D**): Schematic diagram shows the effect of Hsp22 deletion on FA and glucose metabolisms at an older age (red indicates significantly changed proteins in KO vs. WT mice). Data are presented as mean ± SEM. * *p* < 0.05, ** *p* < 0.01 vs. WT. *N* = 4/group. A *t*-test was used.

Next, we investigated the impact of Hsp22 deletion on cardiac metabolism in older age in KO mice, as shown in Figure 6A. Both CPT1a and CPT2 were decreased in the KO mice vs. WT, and PPARα, rather than PPARγ, was reduced in the older KO mice. Reciprocally, two critical transcriptional coactivators that extensively regulate glycolipid metabolism, phosphatidate phosphatase-1 (Lipin1), and Sirtuin 1 (SirT1), were dramatically increased in the older KO mice compared to their age-matched WT littermates (Figure 6B). Lipin1 acts as a crucial regulator for cardiac lipid metabolic homeostasis due to its well-characterized phosphatidate phosphatase (PAP) activity while being a transcriptional coregulator of genes involved in lipid metabolism. SirT1, an NAD+-dependent deacetylase encoded by the SirT1 gene, belongs to the silent information regulator 2 protein family and has been intensively investigated for being an epigenetic regulator that extensively controls energy metabolism.

GLUT1, PKM1/2, and LDHA in glucose metabolism were further decreased in the older KO mice vs. WT. In addition, PFKFB2, a prominent rate-limiting enzyme in glycolysis and gluconeogenesis, which was not significantly different between the young KO and WT mice, became remarkably reduced in the older KO mice vs. WT. Furthermore, PDH, one of the major enzymes responsible for catalyzing mitochondrial pyruvate decarboxylation, was significantly decreased in the older KO mice vs. WT. These data indicate further damage of Hsp22 deletion on glycolysis in the older KO mice, particularly mitochondrial ATP production (Figure 6C). The detrimental effects of Hsp22 deficiency on the cardiac metabolic pathways at an older age are summarized in Figure 6D.

### 3.6. Loss of Hsp22 Increases Age-Related Oxidative Stress in the Hearts of KO Mice

**Figure 7:**
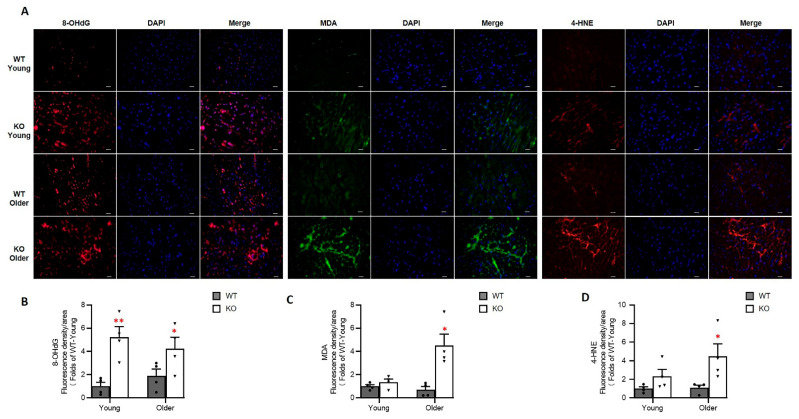
Hsp22 deletion increased oxidative damage in the heart. (**A**): The representative images of oxidative markers in the left ventricular tissues using the immunofluorescent staining with 8-OHdG (red), MDA (green), and 4HNE (red), DAPI staining (blue) for nucleic acid. Scale bars: 20 μm. (**B**–**D**): Quantitated data of the reactive oxygen species (ROS) indicators, including 8-OHdG (**B**), MDA (**C**), and 4HNE (**D**), in the Hsp22 KO and WT mouse hearts at both young and older ages. Data are presented as mean ± SEM. * *p* < 0.05, ** *p* < 0.01 vs age-matched WT. *N* = 4/group. *t*-test was used for the comparison between KO and WT at each age point.

We examined the impacts of Hsp22 deficiency on the cardiac response to oxidative stress during the aging transition. The presence of oxidative stress markers in LV tissues from each group was determined using immunofluorescence as previously described [23], including 8-OHdG, 4HNE, and MDA (Figure 7A). 8-OHdG is an oxidized derivative of deoxyguanosine that serves as a pivotal marker for assessing endogenous oxidative DNA damage. 4HNE, a highly toxic metabolite from reactive oxygen species (ROS) induced lipid peroxidation, is considered a reliable, sensitive marker for oxidative stress. MDA is one of the final products of polyunsaturated fatty acids peroxidation, indicating the formation of free radicals and oxidative stress.

As shown in Figure 7B, compared to the WT mice, 8-OHdG was significantly increased in both the young and older KO mice, indicating increased oxidative DNA damage caused by Hsp22 deletion showing up early in the heart. MDA and 4-HNE were significantly increased in the older Hsp22 KO mice vs. the age-matched WT mice, but not at the young age, suggesting increasingly severe lipid peroxidation in older age (Figure 7C,D).

### 3.7. Loss of Hsp22 Impaires Cardiac Energetics and Activates Cardiac Apoptotic Process in the KO Mice

**Figure 8:**
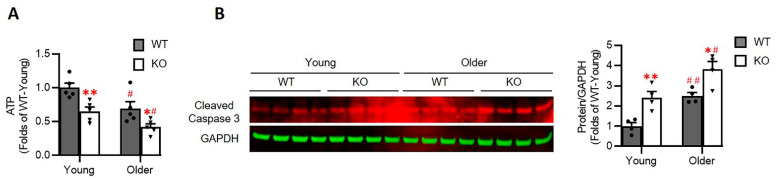
Hsp22 deletion results in a decreased ATP production and activation of Caspase 3 in the heart tissues in KO mice. (**A**): The relative ATP levels in the heart tissue lysis from each group. *N* = 5/group. (**B**). The representative images of western blots and relative values of cleaved caspase 3 in the left ventricular tissues. *N* = 4/group. GAPDH was used as a loading control. Data are presented as mean ± SEM. * *p* < 0.05, ** *p* < 0.01 vs. age-matched WT. ^#^ *p* < 0.05, ^##^ *p* < 0.01 vs. corresponding young mice. *t*-test was used for the comparisons between KO and WT,or young and older.

At last, we investigated whether the alterations in cardiac metabolism observed above affect the cardiac energetics. As shown in Figure 8A, the relative cardiac ATP level was significantly decreased in the hearts of the Hsp22 KO mice than their age-matched WT mice, starting at a young age and progressively deteriorating in older age.

In addition, we also observed that the cleaved Caspase 3, a crucial mediator of programmed cell death (apoptosis), was significantly increased in the heart tissues of the Hsp22 KO mice compared to the age-matched WT mice (Figure 8B), indicating the activation of cardiac apoptosis in the Hsp22 KO mice. This activation was also aggravated in the older mice which consisted of the impairment of ATP products.

## 4. Discussion

Our previous studies found that Hsp22 plays a highly protective role in the stressed heart. The present study provides novel evidence that Hsp22 deletion induces progressive cardiac dilation and dysfunction with increasing age, indicating that Hsp22 is an essential regulator of cardiac function in physiological conditions. In addition, our results also showed that Hsp22 participates in multiple physiological processes involving cardiac autophagy, energy metabolisms, and oxidative response. These findings together indicate a novel, essential role of Hsp22 in cardiac physiology during aging, which was previously uncharacterized.

A highlighted finding of our current study is that Hsp22 deficiency-induced cardiac dilation and dysfunction exhibit a pattern of progressive deterioration. Statistically, Hsp22 deletion did not lead to a significant difference in cardiac morphology and function between the young KO and WT mice but induced progressive LV enlargement and an evident decline in LV contractile function with increasing age, which began in middle age and became worse in older age. These data indicate a chronic detrimental effect of Hsp22 deficiency in the heart. Interestingly, by using a 2D-STE, we detected even earlier signs of cardiac muscle weakness, reflected by significantly reduced LV regional myocardial activity of deformation. The 2D-STE imaging technique measures myocardial deformation better than conventional M-mode ultrasound imaging by capturing segmental tissue motion across multiple planes and axes serially over the cardiac cycle [29,30]. Thus, analyzing 2D-STE images could provide a much more sensitive and specific assessment of the early alteration of cardiac activity. Using this technique, we detected an earlier decline of cardiac contractile activity in the Hsp22 KO mice at a young age. During that time, the M-mode echo was not able to reveal any significant changes in EF and FS yet. These results indicate that the onset of impairment in cardiac muscle activity induced by Hsp22 deletion preceded the decline in cardiac function detected using conventional echo measurements.

Mechanistically, our results revealed the underpinning mechanisms associated with the development of cardiac dilation and dysfunction during the aging transition in the Hsp22 KO mice, involving inhibited BAG3 expression, impaired cardiac autophagy, maladaptive metabolic remodeling, and increased oxidative stress caused by ROS production. It has been shown that BAG3 physically interacts with Hsp22 to form a multi-chaperone complex [31], through which BAG3 mediates Hsp22 stability in cells [32]. A previous study showed that BAG3 deletion could eliminate Hsp22 expression due to structural instability, leading to dilated cardiomyopathy (DCM) [15]. These findings demonstrated the importance of physical interactions between BAG3 and Hsp22. Our study first showed that the reciprocal effect of Hsp22 deletion on BAG3 expression in the heart induced a remarkable reduction in BAG3 in the KO mice in an age-dependent manner, further highlighting the importance of dynamic protein-protein interactions between BAG3 and Hsp22. Moreover, altered BAG3 expression runs concomitantly with the aging-promoted decline in cardiac muscle activity and the development of DCM, suggesting a potential linkage between an Hsp22 deficiency-induced decrease in BAG3 expression and the pathogenesis of DCM in Hsp22 KO mice during early aging.

Although the roles of BAG3 in the heart under physiological conditions have not been fully understood yet, BAG3 mutations have been currently associated with DCM and muscular dystrophy in humans [33], indicating a vital role of BAG3 in cardiac growth and function. It has been reported that BAG3 acts in concert with Hsp22 to induce autophagic degradation of mechanically damaged cytoskeleton components. This process is called chaperone-assisted selective autophagy, essential for muscle maintenance in flies, mice, and humans [12,14,34]. Considering the critical role of BAG3-Hsp22 interaction in regulating selective autophagy within skeletal muscles, we explored the biological function of Hsp22 in cardiac autophagy. Our results demonstrated that Hsp22 deletion promotes cardiac autophagy in young Hsp22 KO mice but significantly decreases in older Hsp22 KO mice. These data imply a dynamic shift in autophagic activity during the development of cardiomyopathy induced by Hsp22 deletion. It is reasonable to assume that a slight increased cardiac autophagy in young Hsp22 KO mice may act as a compensatory response to diminished BAG3 expression to maintain an efficient degradation of damaged proteins. This compensatory response is likely to be associated with the increased expression of the multifunctional Hsp70, which BAG3 binds to form the Hsp70-BAG3 complex while interacting with Hsp22. In old-aged the Hsp22 KO mice whose BAG3 expression continued to decrease but Hsp70 had ceased to increase to compensate for the BAG3 loss, cardiac autophagy started to rapidly decline, leading to an accumulation of damaged proteins and subsequent cytotoxicity, thus aggravating cardiac damage and dysfunction.

Additionally, our results demonstrated that Hsp22 plays an essential role in the age-dependent changes in cardiac metabolism under physiological conditions. Unlike the liver that harbors the largest glycogen storage depot, the heart has very little glycogen available, and it becomes depleted very quickly under hypoxic conditions. Moreover, little gluconeogenesis takes place in the heart. Therefore, the heart derives most of the required ATP via mitochondrial FAO, making the transcriptional regulation of genes encoding the transporters and enzymes involved in FAO particularly important for cardiac energy supply. Our results showed that, at a young age, the loss of Hsp22 decreased cellular FA uptake and the following transport into the mitochondria, which is likely attributable to the reduced ACSL1 activity. This critical enzyme facilitates FA uptake and directs them to β-oxidation, thus maintaining normal lipid trafficking during FA metabolism critical for heart function [35]. In addition, two crucial transcription factors regulating FAO, PGC-1α and PPARγ, were significantly reduced in the hearts of the young Hsp22 KO mice vs. the WT mice. Dynamic interactions between PGC-1α and PPARγ have been reported to maintain FA oxidation rates by promoting the expression of mitochondrial proteins involved in the tricarboxylic acid cycle (TCA cycle), the electron transport chain (ETC), and oxidative phosphorylation. Moreover, PGC-1α also serves as a master transcriptional regulator of mitochondrial biogenesis. Thus, the cooperative functions of PGC-1α and PPARγ are tightly coupled with mitochondrial ATP production, substantially contributing to cardiac energy metabolism homeostasis [36,37]. A reduced PGC-1α expression has been linked to the development of cardiac hypertrophy and failure [38]. PGC-1α deficiency in PGC-1α knockout mice exhibited diminished energy reserves, impaired cardiac contractility [39], and maladaptation to an increased workload [40,41]. The decrease in PGC-1α and PPARγ in Hsp22 KO mice and the resultant cardiac maladaptive remodeling reinforced the essential role of Hsp22 in cardiac energy metabolism. These data together indicate that Hsp22 deletion attenuated cardiac FA metabolism at a young age.

Our result also suggested that Hsp22 mediates cardiac glucose metabolism from an early age. Glucose uptake in cardiomyocytes relies on the translocation of glucose transporters across membranes, followed by glycolytic flux to produce pyruvate, then metabolized to acetyl-CoA by mitochondrial multienzyme complex, such as PDH/PDHK. Our study showed that the loss of Hsp22 could impair glucose metabolism primarily by decreasing the expression of glucose transporters GLUT1 and GLUT4. In addition, three key enzymes that tightly and reciprocally regulate the rates of glycolysis and gluconeogenesis, ALDOA, PKM1/2, and LDHA, were found to be decreased in young Hsp22 KO mouse hearts. Aside from promoting mitochondrial biogenesis, these enzymes mediate diverse cellular functions such as muscle maintenance, regulation of cell shape and motility, striated muscle contraction, actin cytoskeleton organization, and cell proliferation [42,43]. ALDOA has been reported to protect cardiomyocytes against hypoxia/reoxygenation (H/R)-induced myocardial cell apoptosis and oxidative stress [44]. A decrease in these multifunctional enzymes is likely to be a subsequent response to the compromised FA metabolism to re-balancing cellular energy substrate metabolism by controlling critical steps of cytosolic glycolysis and gluconeogenesis in KO mice. Mitochondrial glucose oxidation, on the other hand, has not been significantly affected during this early stage of the aging transition.

We noticed that Hsp22 deficiency impacts cardiac metabolism in an age-dependent manner. For example, FA metabolism was further impaired in the older KO mice, reflected by the decreased expression of more mitochondrial membrane transport proteins and the ligand-activated transcriptional factor PPARα. Notably, glucose metabolism was dramatically impaired in the older KO mice, shown as the reduced glycolytic enzyme expression of PFKFB2 and PDH. PFKFB2 is the heart-specific isoenzyme of PFK2 that is highly responsive to hypoxic-ischemic insults in vivo, such as myocardial infarction (MI)-induced acute ischemia [45]. The decreased PDH, a rate-limiting enzyme that catalyzes pyruvate to acetyl-coenzyme A (acyl-CoA) in mitochondrial, links glycolysis to oxidative phosphorylation [46]. The decrease in PFKFB2 and PDH in the old-aged Hsp22 KO mice indicates a further deterioration in glucose metabolism with aging, possibly contributing to declined cardiac function in the aged heart.

Together, our study demonstrated that Hsp22 is an essential mediator for glucose and FA metabolism. Hsp22 deletion initiated the impairment of FA transport and FAO gene regulation, undermining ATP production from FAO and consequently triggering glycolysis and gluconeogenesis to compensate for energy deficits and decrease the burden of malfunctioning mitochondria. With increasing age, the deleterious effects of Hsp22 deficiency were progressively exacerbated by a stronger and longer-lasting inhibition in glycolysis, particularly mitochondrial pyruvate utilization, which further diminishes the insufficient energy supply and serves as a potential cause of HF in the Hsp22 KO mice. Although metabolic remodeling is a well-characterized hallmark of HF [22,37], the roles of energy metabolism in cardiac aging have not been fully understood. Our results showed that mitochondrial FAO was impaired prior to the development of cardiac dysfunction, suggesting that promoting FA oxidation at an early stage might have a protective role against the progress of cardiometabolic disorder to cardiac functional decline, reducing HF. These early impairments in energy metabolism in KO mouse hearts may also explain the cardiac muscle weakness observed by STE in the young KO mice.

Interestingly, despite a decline in most enzymes involved in cardiac metabolism, two proteins, Lipin1 and SirT1, were significantly increased in the older KO mice but not in the young KO mice. Lipin1 is a bifunctional protein abundantly expressed in the heart serving as a transcriptional coregulator of DNA-bound transcription factors, such as PPARγ and SriT1, to modulate their activities while bearing intrinsic PAP enzymatic activity to catalyze phosphatidate (PA) dephosphorylation during triglyceride biosynthesis. Lipin1 has been reported to coordinate and amplify PPARα–PGC-1α-directed gene expression by forming a dynamic complex with PGC-1α and PPARα, leading to increased FA oxidation. SirT1 is a highly conserved NAD^+^-dependent protein deacetylase that has emerged as a critical regulator of aging and metabolic disease. SirT1 expression was upregulated in the failing heart and the heart of calorie-restricted animals [47]. SirT1 has been correlated with regulating various metabolic pathways, cell survival, and the cellular response to metabolic stressors [48], acting as a nuclear metabolic sensor that detects changes in nutrients and energy metabolism [49]. Cardiac SirT1 overexpression has been reported to retard cardiac aging and upregulate cellular resistance to oxidative stress. Although the functional relevance is not yet clear, the increase in Lipin1 and SirT1 in the old-aged KO mice is likely a compensatory mechanism for mitochondrial dysfunction. However, this hypothesis requires further investigation and confirmation.

We also found that Hsp22 deletion results in an age-dependent accumulation of oxidative cytotoxic products in the heart, leading to cardiac oxidative damage in the older age in Hsp22 KO mice. Several potential mechanisms may be involved in the oxidative stress caused by the deficiency of Hsp22 in the heart. First, the loss of Hsp22 reduced cardiac BAG3 expression, resulting in impaired cardiac autophagy and subsequent accumulation of damaged DNA and proteins in cardiomyocytes, increasing ROS products. Secondly, the loss of Hsp22 disturbed cardiac metabolism, particularly in mitochondrial FAO, leading to an excessive accumulation of lipid intermediates and potential cardiac lipotoxicity. Thirdly, the decline in mitochondrial function further aggravated the ill-balanced energy supply and markedly increased ROS production in the heart. Lastly, cardiac dysfunction inexorably hinders the normal cellular processing of oxygen and nutrition of the heart tissues, exacerbating cardiac oxidative stress.

The heart is a highly energy-depended organ and relies on the ATP supply to maintain its continuous mechanical work. Our results indicate that a loss of Hsp22 results in an impairment in ATP generation, which may be partially caused by the alterations in cardiac metabolism. Impaired cardiac energetics not only affect cardiac function directly but may also affect cell growth and survival by inducing other processes, such as oxidative stress, cell apoptosis, and structural remodeling, leading to cardiac dysfunction and dilation.

## 5. Conclusions

**Figure 9:**
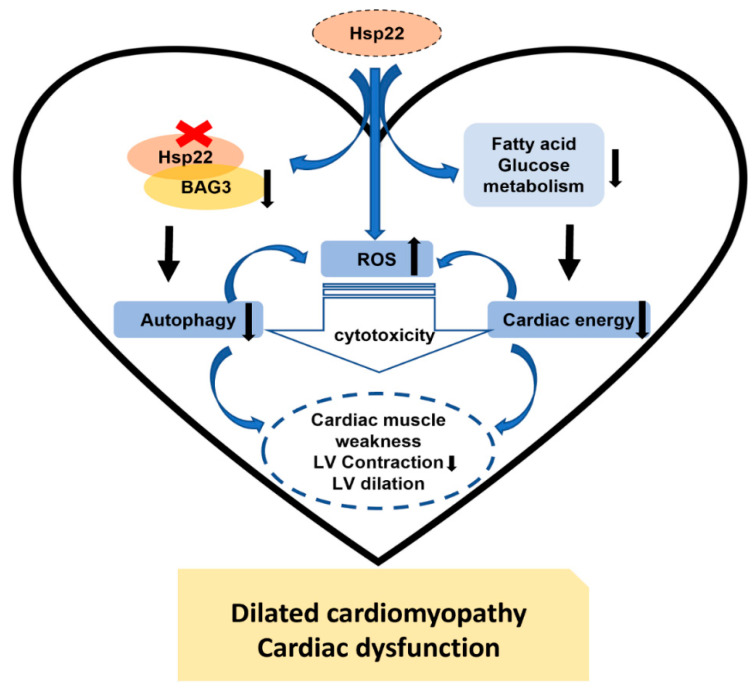
Summary of the findings. The results from this study indicate that Hsp22 deficiency interferes with multiple biological functions in the heart during the aging transition, involving the down-regulation of BAG3 and impairment of fatty acid and glucose metabolism starting at a young age, which impacts cardiac muscle activity, and impairs cardiac autophagy and increases oxidative damage in older age, inducing a progressive cardiac dilation and lower left ventricular contractility, leading to dilated cardiomyopathy and cardiac dysfunction in the aging heart.

In summary, as illustrated in Figure 9, our study demonstrated that Hsp22 plays an essential role in maintaining cardiac function under physiological conditions. Deleting Hsp22 induced an age-dependent cardiac dilation and myocardial dysfunction via a comprehensive mechanism involving impaired cardiac autophagy, metabolic remodeling, oxidative response, resulting in cardiac energetic deficiency and cell death. Our study also provided novel insights into the diverse roles of Hsp22 in regulating heart physiology and the potential interaction among autophagy, energy metabolisms, and oxidative injury in the aging heart, which may also be involved in the pathogenic pathway of heart failure.

## Figures and Tables

**Table 1:** Information of the primary antibodies used in this study.

Antibody	Application	Supplier	Catalog Number	Dilution
8-OHdG	IFC	Santa cruz	sc-66036	1:200
MDA	IFC	Invitrogen	MA5-27559	1:200
4HNE	IFC	Invitrogen	MA5-27570	1:200
GLUT1	WB	Cell signaling	#12939	1:5000
GLUT4	WB	Abcam	ab654	1:5000
HK1	WB	Cell signaling	#2024	1:5000
HK2	WB	Cell signaling	#2867	1:5000
PFKFB1	WB	Abcam	ab155564	1:5000
PFKFB2	WB	Cell signaling	#13045	1:5000
PFKM	WB	Abcam	ab154804	1:10,000
ALDOA	WB	Cell signaling	#8060	1:5000
PKM1/2	WB	Cell signaling	#3190	1:5000
LDHA	WB	Cell signaling	#3582	1:5000
p-PDH	WB	Abcam	ab177461	1:5000
PDH	WB	Cell signaling	#3205	1:5000
PDHK1	WB	Cell signaling	#3820	1:5000
CD36	WB	Abcam	ab133625	1:10,000
ACSL1	WB	Cell signaling	#9189	1:5000
Phospho-AMPKα	WB	Cell signaling	#2535	1:5000
AMPKα	WB	Cell signaling	#2532	1:5000
CPT1a	WB	Abcam	ab234111	1:10,000
CPT2	WB	Abcam	ab181114	1:10,000
Lipin1	WB	Cell signaling	#14906	1:2000
SirT1	WB	Cell signaling	#2496	1:2000
PGC1α	WB	Invitrogen	PA5-38021	1:10,000
PPAR γ	WB	Santa cruz	sc-7196	1:2000
BAG3	WB	Proteintech	10599-1-AP	1:5000
Hsp70	WB	Cell signaling	#4872	1:5000
SQSTM1/p62	WB	Cell signaling	#5114	1:5000
LC3A/B	WB	Cell signaling	#12741	1:5000
Cleaved casp-3	WB	Cell signaling	#9661S	1:10,000
Hsp22	WB	Handmake	Handmake	1:10,000
β-tubulin	WB	Sigma-Aldrich	T8328	1:10,000
GAPDH	WB	Cell signaling	#97166	1:20,000

## Data Availability

The data supporting reported results are provided as requested in a separate file.

## Supplementary Materials

The following are available online at https://www.mdpi.com/article/10.3390/antiox10101550/s1, Figure S1: The expression of Hsp22 and its associated proteins progressively altered during the aging transition.
