# Supplementary material for: Hsp22 Deficiency Induces Age-Dependent Cardiac Dilation and Dysfunction by Impairing Autophagy, Metabolism, and Oxidative Response
Source: Antioxidants (Basel). 2021 Sep 29;10(10):1550. doi: 10.3390/antiox10101550 (PMC8533440; doi:10.3390/antiox10101550)
Supplement: Supplementary file 1 [file antioxidants-10-01550-s001.zip › antioxidants-1347972-supplementary.pdf]

Supplemental Data

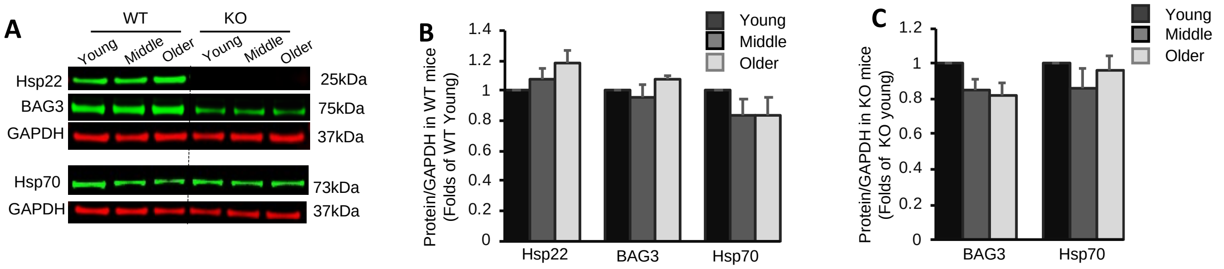

Figure S1. The expression of Hsp22 and its associated proteins progressively altered during the aging transition. A. The representative western blotting images of Hsp22, BAG3 and Hsp70 in mouse heart tissues at different ages (young, middle age and elder) in WT and Hsp22 KO mice. GAPDH was used as loading control. **B.** The relative values of Hsp22, BAG3 and Hsp70 proteins in WT mice in each age group**.** ***C.*** The relative values of BAG3 and Hsp70 proteins in Hsp22 KO mice during aging transition. N=3/group for B and C. The expressions of corresponding proteins were normalized to GAPDH and presented by the fold of the young age mice respectively in WT and KO mice.
